# The Effect of Inappropriate Calibration: Three Case Studies in Molecular Ecology

**DOI:** 10.1371/journal.pone.0001615

**Published:** 2008-02-20

**Authors:** Simon Y. W. Ho, Urmas Saarma, Ross Barnett, James Haile, Beth Shapiro

**Affiliations:** 1 Department of Zoology, University of Oxford, Oxford, United Kingdom; 2 Department of Zoology, Institute of Ecology and Earth Sciences, University of Tartu, Tartu, Estonia; 3 Estonian Biocentre, Tartu, Estonia; 4 Department of Biology, University of York, York, United Kingdom; University of Kent, United Kingdom

## Abstract

Time-scales estimated from sequence data play an important role in molecular ecology. They can be used to draw correlations between evolutionary and palaeoclimatic events, to measure the tempo of speciation, and to study the demographic history of an endangered species. In all of these studies, it is paramount to have accurate estimates of time-scales and substitution rates. Molecular ecological studies typically focus on intraspecific data that have evolved on genealogical scales, but often these studies inappropriately employ deep fossil calibrations or canonical substitution rates (e.g., 1% per million years for birds and mammals) for calibrating estimates of divergence times. These approaches can yield misleading estimates of molecular time-scales, with significant impacts on subsequent evolutionary and ecological inferences. We illustrate this calibration problem using three case studies: avian speciation in the late Pleistocene, the demographic history of bowhead whales, and the Pleistocene biogeography of brown bears. For each data set, we compare the date estimates that are obtained using internal and external calibration points. In all three cases, the conclusions are significantly altered by the application of revised, internally-calibrated substitution rates. Collectively, the results emphasise the importance of judicious selection of calibrations for analyses of recent evolutionary events.

## Introduction

Evolutionary time-scales estimated from molecular data form the foundation for a diverse range of molecular ecological studies, including those of biogeography, speciation, conservation genetics, and population biology. Molecular chronologies allow us to examine correlations between evolutionary events and palaeoclimatic phenomena, such as glaciations and sea level changes [1]–[3], or biotic factors, including faunal and floral changes and human migration [4]–[6]. For conservation purposes, we can measure the phylogenetic distinctiveness of a species or the antiquity of a specific population [7], [8], while past and present effective population sizes can be inferred in studies of population biology [9], [10]. All of these studies depend on accurate estimates of molecular time-scales and substitution rates which, primarily due to methodological limitations, have been made with varying degrees of rigour in the past.

Estimating time-scales from genetic data is laden with methodological obstacles. One of the chief difficulties is the selection of an appropriate calibration [11]–[14], which is necessary for converting measures of genetic divergence into units of absolute or geological time. Calibrating information can be incorporated into an analysis in one of several ways [15], of which the most widely used are: (i) fixing the age of a phylogenetic divergence event on the basis of independent palaeontological, archaeological, or biogeographic data; and (ii) importing a substitution rate obtained from independent data. A third calibration method is the inclusion of heterochronous sequences of known age, such as ancient DNA sequences extracted from radiocarbon-dated samples [16], [17].

The fossil record has played a key role in calibrating molecular estimates of evolutionary rates and divergence times in the tree of life [18]. This role has expanded in recent years due to advances in phylogenetic methods [15], [19]. Ecological studies, however, often investigate evolutionary processes within species, such as the timing of dispersal, migration, or extinction events [4], [20]. These occur on genealogical time-scales rather than the longer phylogenetic time-scales typically associated with fossil calibrations. Using deep fossil calibrations in ecological studies can present a methodological problem because different stages of the substitution process are being observed over genealogical and phylogenetic time-scales [21]. When sequences are taken from individuals within a population or species, the differences among them represent segregating sites or polymorphisms, many of which are transient and will be removed by genetic drift or purifying selection [21]–[24]. In contrast, differences between sequences taken from different species represent past fixations (substitutions).

This is, of course, a simplistic portrayal of the situation, as the species boundary is often indistinct and the two scales are directly linked because mutation and substitution are aspects of the same population genetic processes. For example, the observed genetic variation within a species can be inflated by the presence of ancestral polymorphisms that have been inherited from the parent species [25].

Nevertheless, it is apparent that considerable disparities can result between long-term substitution rates and instantaneous mutation rates, with direct and indirect estimates of the latter frequently yielding high values [26]–[28]. This disparity can be magnified by saturation at mutational hot spots [29], which can obscure the presence of past polymorphisms and cause long-term rates to be underestimated if inadequate corrections are applied.

**Figure 1:**
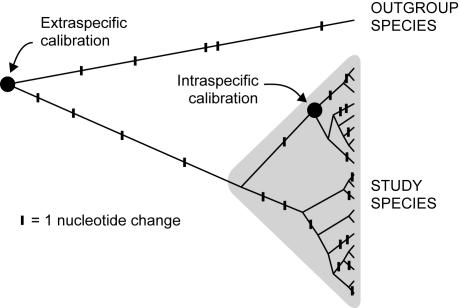
Phylogenetic tree illustrating the impact of using extraspecific and intraspecific calibration points. The tree shows the locations of nucleotide changes (small vertical bars). The nucleotide changes within the study species represent segregating sites, some of which will be fixed in the long term, but most of which will be removed by drift or selection. The changes between the study species and outgroup species represent substitutions. If an estimate of the evolutionary rate is calibrated using an external calibration point, such as the split between the study and outgroup species, then the intraspecific rate will be underestimated. This will lead to an overestimation of times to divergence, including the age of the most recent common ancestor of the study species.

In a phylogenetic analysis, transient polymorphisms manifest themselves as excess nucleotide changes on the branches near the tips of the tree (Figure 1) [30], [31]. They disappear from the population through time, so that the deeper a branch is in the tree, the fewer transient polymorphisms it will carry. When multiple species are compared within a single tree, the majority of nucleotide changes observed along the deepest branches, including the branch between the study species and outgroup, are likely to represent substitutions. Consequently, deeper calibration points will lead to slower observed rates [21], [23]. This can lead to considerable overestimates of times to divergence, particularly when slow substitution rates are obtained from interspecific comparisons in a phylogenetic context and then extrapolated to population-level data [21], [32].

We believe that the inappropriate use of extraspecific or external calibration points may have misled a substantial number of molecular ecological studies. Here we present three detailed case studies in which the conclusions are significantly altered by using intraspecific, internal calibration points.

## Results and Discussion

### Avian Speciation in the Late Pleistocene

In Eurasia and North America, the Pleistocene was a time of significant fluctuations in climate and the distribution of habitats. Cyclical glacial advance and retreat produced dramatic changes in local environmental conditions over the past 250 kyr [33], leading to the view that these changes were conducive to an increased rate of avian speciation, a concept embodied in the “Late Pleistocene Origins” (LPO) hypothesis [for a recent review, see 1]. This hypothesis readily lends itself to testing because it makes specific predictions about divergence times, which can be estimated from molecular data. A study of mitochondrial DNA from avian sister species found that divergences occurred prior to the Pleistocene [34]. On the basis of this evidence the authors suggested that the speciation events were associated with early glacial expansion in the Northern Hemisphere around 2.4 Myr before present (BP). In contrast, an analysis of diverging conspecific populations or ‘phylogroups’, which were interpreted as reflecting incipient speciation, supported a more recent time-scale for avian diversification [35]. The debate has continued in numerous subsequent studies [36]–[42].

In nearly all of these studies, the authors collected a series of genetic divergence estimates, then divided the distances by a known substitution rate in order to produce an estimate of the time since divergence. The reliability of such estimates depends absolutely on the accuracy of the imported rate. Without exception, the substitution rate used in tests of the LPO hypothesis has been the ‘traditional’ mitochondrial rate of 0.01 substitutions per site per million years (subs/site/Myr), which has long been adopted as a standard in avian molecular studies [43], [44]. There are several reasons why the use of this rate may be questionable. First, the rate is not universally applicable among different avian taxa, with considerable rate heterogeneity detected among lineages [45], [46]. Second, the rate was calculated in a phylogenetic context, whereas the study of incipient species (and perhaps recently diverged species) is a genealogical issue [32]. Studies of intraspecific data from birds have generally yielded faster substitution rate estimates [23], [47], peaking at a rate of 0.95 subs/site/Myr for the mitochondrial hypervariable region 1 in Adélie penguins [48]. To investigate the impact of employing these higher rates in investigations of the LPO hypothesis, we re-analysed sets of genetic distances made in published studies.

Using a rate of 0.01 subs/site/Myr, only three of the 22 species had phylogroup divergences occurring within the past 250 kyr, whereas three species had Pliocene (>1.8 Myr) ages (Table 1). By applying a higher, revised rate of 0.075 subs/site/Myr, obtained from an internally-calibrated analysis of amakihi subspecies [22] (see Materials and Methods), we found that the evidence shifted significantly towards support for late Pleistocene divergences. Only three species yielded phylogroup divergences exceeding 250 kyr. Our date estimates do not take into account the uncertainty associated with the imported, revised rate, but if the highest rate implied by the upper 95% credibility limit on the original rate estimate [0.111 subs/site/Myr; 22] is applied to the data, then all of the resulting divergence time estimates fall within the past 250 kyr.

**Table 1:** Divergence time estimates for conspecific phylogroups from 22 bird species

Family^a^	Species	Genetic distance^b^	Divergence time estimate^c^ (Myr)	
			Rate = 0.01 subs/site/Myr	Rate = 0.075 subs/site/Myr
Paridae	*Poecile gambeli*	5.442	*2.721*	*0.363*
Parulinae	*Wilsonia pusilla*	5.188	*2.594*	*0.346*
Certhiidae	*Polioptila caerulea*	4.008	*2.004*	*0.267*
Turdidae	*Catharus guttatus*	3.397	*1.698*	0.226
Vireonidae	*Vireo gilvus*	3.228	*1.614*	0.215
Paridae	*Poecile carolinensis*	2.900	*1.450*	0.193
Emberizinae	*Passerella iliaca*	2.858	*1.429*	0.191
Parulinae	*Dendroica petechia*	2.377	*1.189*	0.158
Turdidae	*Catharus ustulatus*	1.420	*0.710*	0.095
Parulinae	*Geothlypis trichas*	1.033	*0.517*	0.069
Mimidae	*Toxostoma redivivum*	0.824	*0.412*	0.055
Paridae	*Baeolophus inornatus*	0.781	*0.390*	0.052
Emberizinae	*Melospiza melodia*	0.708	*0.354*	0.047
Sylviidae	*Chamaea fasciata*	0.704	*0.352*	0.047
Tyrannidae	*Empidonax traillii*	0.614	*0.307*	0.041
Paridae	*Baeolophus ridgwayi*	0.558	*0.279*	0.037
Fringillidae	*Carduelis hornemanni*	0.551	*0.275*	0.037
Emberizinae	*Calcarius lapponicus*	0.550	*0.275*	0.037
Vireonidae	*Vireo solitarius*	0.532	*0.266*	0.035
Picidae	*Picoides dorsalis*	0.457	0.228	0.030
Emberizinae	*Spizella breweri*	0.291	0.146	0.019
Emberizinae	*Zonotrichia leucophrys*	0.231	0.116	0.015

a All taxa are members of Order Passeriformes, with the exception of Picidae (Order Piciformes).

b Gamma-corrected distance estimated by Weir and Schluter [42].

c Divergence times older than 250 kyr are italicised.

Our results should be interpreted cautiously because of the dubious applicability of a single substitution rate to different avian species. Collectively, however, our analyses demonstrate that the published molecular evidence used to challenge the LPO hypothesis is weakened by the application of a revised rate. This does not necessarily signify that glacial cycles provided conditions that were exceptionally suitable for allopatric speciation among birds, and it remains unclear whether the tempo of avian speciation in the Pleistocene was actually elevated in comparison to preceding geological periods [36], [39], [40]. Nevertheless, the sensitivity of tests of the LPO to the assumed substitution rate emphasises the importance of selecting an appropriate calibration. This concern also applies to similar studies of late Pleistocene divergences among other organisms [49].

### Demographic History of the Bowhead Whale

The bowhead whale (*Balaena mysticetus*) was subjected to intensive commercial exploitation from the 17th to early 20th centuries. During this period, over 90,000 individuals were taken from the Spitsbergen stock alone [50]. This stock remains critically endangered, while two of the other four designated stocks are endangered. Bowhead whales are exceptionally long-lived, with a maximum longevity well in excess of 100 years, and appear to reach sexual maturity at around 25 years of age [51]. These observations suggest a long generation time, which presents obvious difficulties for population management and stock recovery. For these reasons, the demographic history of this species is of significant ecological, commercial, and conservational interest.

An analysis of modern DNA samples from the mitochondrial control region of 98 individuals found that the most recent common ancestor (MRCA) of modern bowhead whales lived around 267 kyr ago [9]. The estimate was calibrated using a value of 3.4 Myr for the age of the split between bowhead whales (*Balaena*) and right whales (*Eubalaena*). This calibration point was informed by the fossil record, which is relatively sparse for balaenids; the oldest fossil that can be confidently assigned to either the *Balaena* or *Eubalaena* lineage dates from the Pliocene (5.2 to 2.6 Myr BP). Several lines of evidence, however, suggest a more protracted evolutionary history for the two lineages. First, the Pliocene *Balaena* fossils appear to be highly derived [52]. Second, there is a conspicuous hiatus between the Pliocene fossils and the most ancient fossil representative of Balaenidae, *Morenocetus parvus*, which dates from the earliest Miocene around 20–23 Myr BP [52]. Independent support for a deeper split between the two genera was provided by analysis of whole mitochondrial genomes, which produced an age estimate of 17 Myr [53].

The palaeontological uncertainty over the age of the bowhead-right whale split, the possible antiquity of the divergence event, and the external nature of this calibration all argue against its use in studying the recent demographic history of bowhead whales. To investigate the effect of calibration choice, we use ancient DNA sequences to infer an internally-calibrated evolutionary time-scale.

**Figure 2:**
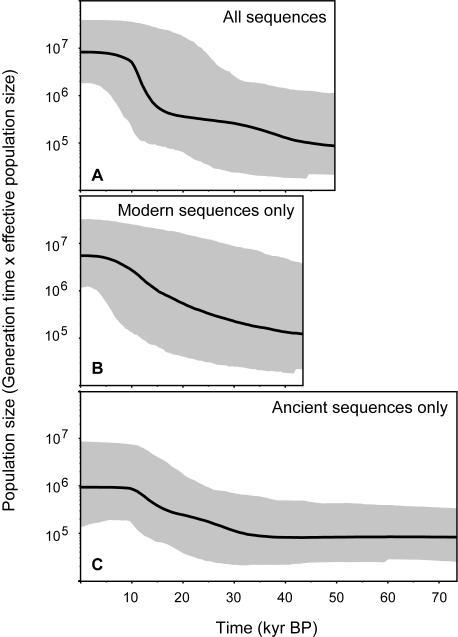
Bayesian skyline plots showing the recent demographic history of bowhead whales, estimated using phylogenetic analysis of three alignments of the mitochondrial control region: (a) combined alignment of 68 modern haplotypes and 99 radiocarbon-dated, ancient DNA sequences; (b) modern sequences only; and (c) ancient sequences only. All three plots are drawn to the vertical and horizontal scales.

We reconstructed the demographic history of bowhead whales using Bayesian skyline plot (BSP) analysis, which generates a plot of the estimated effective population size through time [5] (see Materials and Methods). We found evidence of a population expansion in the late Pleistocene (Figure 2). This pattern emerges for three data configurations (modern only, ancient only, and combined modern and ancient), suggesting that it is not an exclusive artefact of the chronologically heterogeneous sampling of ancient DNA sequences. The age of the MRCA of all individuals was estimated at 153 kyr BP, with a 95% highest posterior density (HPD) of 49.6–294 kyr BP. The estimated timings of the MRCA and population expansion of bowhead whales are more recent than those inferred by Rooney et al. [9].

The estimated substitution rate was 0.159 subs/site/Myr (95% HPD: 0.051–0.272 subs/site/Myr). Although this is lower than other rate estimates obtained in some ancient DNA studies [for lists], [see 22,54], it is significantly higher than the substitution rates obtained using fossil-based point calibrations of either 3.4 or 17 Myr for the bowhead-right whale split, which yield estimates of 0.017 subs/site/Myr (95% HPD: 0.009–0.0029 subs/site/Myr) and 0.0034 subs/site/Myr (95% HPD: 0.0019–0.0057 subs/site/Myr), respectively. By using these two rate estimates to specify a prior on the substitution rate in analyses of the modern bowhead whale sequences, the age of the MRCA of all individuals is estimated to be 1.1 Myr BP (95% HPD: 0.497–1.88 Myr BP) and 4.12 Myr BP (95% HPD: 2.07–6.39 Myr BP), respectively. These are substantially older than the estimate of 150 kyr obtained in the BSP analyses calibrated by radiocarbon dates.

The results suggest that previous estimates of the evolutionary time-scale of bowhead whales have been misled by an inappropriate fossil calibration, the effect of which has been to produce overestimates of times to coalescence. Our re-analysis suggests that population expansion in bowhead whales occurred relatively recently and that short time periods may suffice for the generation of appreciable levels of genetic diversity. This has important consequences for studies of conservation genetics, many of which have relied on substitution rates obtained by adopting relatively deep calibration points.

### Pleistocene Biogeography of the Brown Bear

The last few years have seen a proliferation of large data sets that sample individuals from a population over thousands to tens of thousands of generations. These data enable the direct testing of hypotheses concerning the relationship between past and present distributions of species and populations [20], and the impact of environmental events on the phylogeography of a species [4], [55]. In these studies, it is evident that without accurate estimates of substitution rates, there is a risk of misidentifying causal environmental factors for inferred demographic changes.

**Figure 3:**
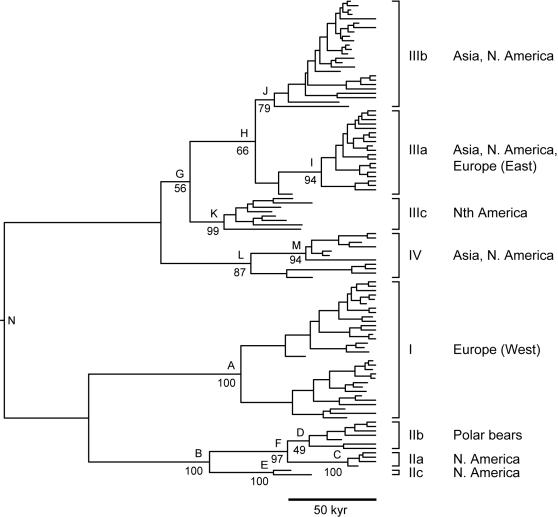
Maximum clade credibility tree from Bayesian analysis of mitochondrial control region sequences from 56 modern and 51 ancient brown bear samples, with a time scale calibrated using the radiocarbon dates of the ancient sequences. Major clades and their geographic localities are given. Posterior probabilities are given for the nodes A-N, which are referred to in the text and in Table 2.

Brown bears (*Ursus arctos*) present an interesting illustration of this point. Modern brown bear populations are distributed throughout Europe, Asia, and North America, and exhibit significant maternally-linked phylogeographic structure across this range [20], [56]–[58] (see tree topology in Figure 3). The modern distribution of brown bears is believed to be the consequence of post-glacial expansion from local refuges, and to have remained relatively stable since this expansion [3], [57], [59]–[61]; however, a more recent study of ancient DNA found a more complex phylogeographic history for Western European brown bears [62]. Although this work did not entirely exclude the classic refuge scenario, it insinuated that past refugium theories might be oversimplifications [62]. Nevertheless, ancient DNA studies have revealed that the modern European distribution of brown bear lineages differs from that prior to the last glacial maximum [55], whereas in North America four distinct periods of constant population structure could be identified over the last 50 kyr, interposed by brief periods of rapid demographic change [20]. These findings suggest that brown bears in both Europe and North America experienced frequent local extinctions and range expansions during the climatically volatile late Pleistocene.

Saarma et al. [3] investigated the possibility that the present distribution of bears in northern Europe is the result of post-glacial expansion from a refuge in the West Carpathian mountains. Using a substitution rate estimated from radiocarbon-dated North American brown bears [20], they estimated that the MRCA of the Eastern lineage (node I, Figure 3) existed 24 kyr BP (95% HPD: 6–50 kyr BP), with dates of 67 kyr BP (95% HPD: 20–131 kyr BP) for the Western lineage (node A) and 174 kyr BP (95% HPD: 61–314 kyr BP) for all European bears (node N). The estimates came with wide 95% HPD intervals, but were consistent with recent expansions of the Eastern and Western lineages and a mid-late Pleistocene MRCA for all brown bears.

These relatively recent estimates for the origin of the European brown bear lineages stand in contrast to the more ancient estimates of Hofreiter *et al*. [63], which were calibrated using the split between brown bears and their sister species (cave bears; *Ursus speleaus*) at 1.2–1.7 Myr [64], [65]. This calibration put the ages of the MRCAs for the two European lineages of brown bears during the mid-early Pleistocene: 640 kyr BP (95% CI: 290–1,390 kyr BP) for the Eastern lineage and 350 kyr BP (95% CI: 150–790 kyr BP) for the Western lineage.

The disparity between the two sets of estimates illustrates the difference between internal and external calibration, but the estimates are difficult to compare directly because they were made using different approaches. To investigate this further, we performed a Bayesian phylogenetic analysis of an alignment of 107 brown bear sequences using two separate modes of calibration: (i) internal calibration using 31 radiocarbon-dated ancient DNA samples; and (ii) importation of a rate estimated using the external cave-brown bear split.

The inferred tree topology was identical for both analyses (Figure 3). Internal calibration produced a rate estimate of 0.390 subs/site/Myr (95% HPD: 0.264–0.526 subs/site/Myr), whereas external calibration yielded a slower rate of 0.061 subs/site/Myr (95% HPD: 0.045–0.075 subs/site/Myr), with correspondingly older MRCA age estimates (Table 2).

**Table 2:** Bayesian age estimates for nodes labelled in Figure 3

Node	Clade	Age estimate (kyr)			
		External calibration		Internal calibration	
		Mean	95% HPD	Mean	95% HPD
A	I	415	198–646	74	45–109
B	II	415	209–659	94	62–133
C	IIa	72	17–149	16	10–26
D	IIb	208	55–398	43	19–73
E	IIa and IIb	319	115–548	51	26–85
F	IIb and IIc	112	50–197	58	48–72
G	III	525	307–793	111	79–148
H	IIIa and IIIb	374	89–741	35	11–75
I	IIIa; modern only	362	67–735	32	9–74
J	IIIb	380	168–620	65	36–110
K	IIIc	260	147–394	86	67–108
L	IV	458	167–755	75	40–130
M	IV; modern US only	164	49–323	31	7–59
*N*	*All brown bears*	*1,159*	*745–1,622*	*211*	*143–295*

Both of our age estimates for the MRCA of the Western lineage (clade I) were similar to those made by previous studies [3], [20]. The internally-calibrated date estimate suggests an MRCA during the previous (Weichselian) glacial period, but the 95% HPD of the externally-calibrated estimate encompasses several glacial and interglacial periods, prohibiting a detailed correlation with environmental change. The evolutionary time-frame of the Eastern European clade, which makes up part of clade IIIa, is less clear. It is interesting that there is no distinct subdivision between European and Alaskan brown bears in clade IIIa, which could be interpreted as evidence for recent expansion into Alaska and Europe from a single source population, although further sampling of late Pleistocene brown bears in Europe and Asia will be required to test this hypothesis.

Brown bears are believed to have established the first populations in North America around 50–75 kyr BP, during the early and middle parts of the last (Wisconsinan; equivalent to the Weichselian in Europe) glaciation [66], [67]. Age estimates for clades II, III, and IV (Table 2) therefore support previous claims that the modern lineages were established prior to the initial colonization of North America [57], [61], most probably during the previous interglacial/glacial transition. Conversely, the MRCA of bears from the Alaskan ABC islands (clade IIa, node C) existed around 16 kyr BP (95% HPD: 10–26 kyr BP), consistent with the timing of the most recent glacial/interglacial transition.

The age of the MRCA of polar bears (node D) is estimated at 43 kyr BP (95% HPD 19–73 kyr BP), and the divergence between polar bears and ABC islands bears (node F) is estimated to have occurred about 58 kyr BP (95% HPD: 48–72 kyr BP). Despite the internal calibration with a multitude of dated tips, the 95% HPD intervals for these estimates are wide, indicating only that polar bears diverged from other brown bears some time after the warmest part of the Wisconsinan glacial period, but lacking the power to identify any specific environmental event or geographic location.

This example illustrates the impact of inappropriate calibration on inferences made from molecular data. It also demonstrates that it can be difficult to correlate demographic data inferred from phylogenies with large-scale environmental fluctuations, due to the considerable statistical uncertainty that is often associated with intraspecific estimates of divergence times. It is possible to reduce this uncertainty by increasing sample size and alignment length, but the most powerful approach might involve the use of molecular data in conjunction with alternative methods, such as radiocarbon dating to find terminal occurrences.

### Concluding Remarks

Our three case studies demonstrate that the findings of molecular ecological studies can be altered significantly by the choice of calibration. Our results show that divergence time estimates can change by an order of magnitude when relatively recent, internal calibration points are used (Table 3). Deep calibration points, particularly those based on the fossil record, lead to inferred evolutionary scenarios that are drastically different from those implied by internal, intraspecific calibrations. The large disparity between estimates obtained by internal and external calibration also highlights the importance of judicious selection of calibrations for recent evolutionary events, even if one does not subscribe to the hypothesis of time-dependent rate estimates [68], [69].

**Table 3:** Summary of estimates made using internal and external calibrations for the three data sets presented in this study

Evolutionary event	Age estimate (kyr)			
	External calibration		Internal calibration	
	Mean	95% HPD	Mean	95% HPD
Common ancestor of bowhead whales				
External calibration = 3.4 Myr	1,140	497–1,880	154	50–295
External calibration = 17 Myr	4,123	2,071–6,386		
Common ancestor of brown bears	1,159	745–1,622	211	143–295
Mean divergence time of conspecific avian phylogroups	878	116–2,721^a^	117	15–363^a^

a Range of 22 divergence time estimates

Intraspecific calibrations can be obtained in one of several ways. As illustrated in the bowhead whale and brown bear examples above, the radiocarbon ages of ancient samples can be used as calibrations on the tips of the tree. Alternatively, internal calibrations can be obtained from biogeography [70], [71], although such calibrations can be difficult to interpret and specify correctly [12], [68]. The fossil record will rarely be able to offer intraspecific calibrations unless substantial diagnostic variation exists within a species, for example among subspecies or conspecific populations. Frequently, however, intraspecific calibration data are unavailable for many data sets, leaving the less desirable option of importing a substitution rate estimated from another (preferably related) species.

A considerable disadvantage of intraspecific calibrations is that they will often produce date estimates with a larger degree of uncertainty than those made using external calibrations. There are two reasons for this: (i) external calibrations are often placed at the root of the tree; and (ii) the removal of outgroup species reduces the information in the data set. Moreover, molecular date estimates can come with substantial uncertainty, so that it can be difficult to draw strong conclusions from molecular date estimates if the associated estimation error is taken into account. Therefore, a reduction in precision appears to be the consequence of using intraspecific calibrations, but the improvement in accuracy should come as a worthwhile recompense.

## Materials and Methods

### Avian Speciation in the Late Pleistocene

In order to test the hypothesis of late Pleistocene origins for birds, we obtained a set of pairwise genetic distances estimated from avian mitochondrial cytochrome *b* by Weir and Schluter [42]. Of these genetic distances, we restrict our re-analysis to the 22 measured between conspecific phylogroups from species with a geographic range extending above 40°N, the approximate boundary of Pleistocene glaciation in North America [72] (Table 1). To convert the distance estimates to time durations, two different substitution rates were applied. The first rate was 0.01 subs/site/Myr, which was used in the original study and corresponds to the canonical 1% rate in birds. The second rate was 0.075 subs/site/Myr, based on an analysis of cytochrome *b* sequences from two subspecies of amakihi from Hawaii and Maui [22]; this is one of the few internally-calibrated rate estimates available for avian cytochrome *b*.

### Demographic History of the Bowhead Whale

To investigate the demographic history of modern bowhead whale populations, we assembled a data set comprising both ancient and modern DNA sequences. Ancient DNA (aDNA) sequences (ages from ranging from 30 years to 51 kyr) from the mitochondrial control region of 99 bowhead whales were obtained from the study by Borge et al. [73]. Modern DNA sequences for 68 haplotypes were obtained from the study by Rooney et al. [9]. Demographic history was inferred from these data using Bayesian skyline plot [BSP; 5] analyses, performed using *BEAST* 1.4 [74] with the substitution model selected by comparison of Akaike Information Criterion values. Three BSP analyses were performed: (i) aDNA sequences only; (ii) modern DNA sequences only; and (iii) ancient and modern DNA sequences combined. For all three analyses, 10 group sizes were used for the BSP. Posterior distributions of parameters were investigated using Markov chain Monte Carlo (MCMC) analysis, with samples from the posterior drawn every 10,000 steps over a total of 50,000,000 steps, with the first 10% discarded as burn-in.

In the two analyses using aDNA sequences, time-scales were calibrated using the radiocarbon dates of the 99 ancient samples. The analysis of modern DNA sequences was calibrated by specifying a normally-distributed prior on the substitution rate (mean 0.149 subs/site/Myr, standard deviation 0.052 subs/site/Myr), based on the value estimated in the aDNA analysis. The modern and ancient DNA data sets were obtained from different stocks (Bering-Chukchi-Beaufort Seas and Spitsbergen, respectively), but there appears to be little molecular or morphological evidence for the discreteness of any of the five designated stocks of bowhead whales [73], [75], [76].

To estimate the average substitution rate across balaenids, control region sequences were obtained from GenBank for the three extant species of right whale (*Eubalaena japonica*, *E. australis*, and *E. glacialis*). These were aligned with the corresponding sequence from *Balaena mysticetus* haplotype A. The alignment was analysed with *BEAST* using fossil calibrations. The oldest fossil representative of either the *Eubalaena* or *Balaena* lineage dates from the Pliocene (1.8–5.3 Myr BP) [77]. Rooney et al. [9] summarised this information by taking a mean value of 3.4 Myr, which we have adopted for comparative purposes. Following the results of Sasaki et al. [53] and in view of the fossil hiatus for balaenids during the Miocene, we repeated the analysis using a calibration age of 17 Myr for the *Balaena*-*Eubalaena* split. In the MCMC analysis, samples were drawn from the posterior every 10,000 steps over a total of 10,000,000 steps, with the first 10% discarded as burn-in. All input files are available upon request from the corresponding author.

### Pleistocene Biogeography of the Brown Bear

To investigate the timing of brown bear movements in the Pleistocene, we collected ancient and modern DNA for phylogenetic analysis. Mitochondrial control region sequences from 51 ancient brown bears were obtained from published sources [20], [55], [62], [78]. In addition, we obtained all 56 modern haplotypes that were available on GenBank, including only the sequences that spanned the same 195 bp stretch as the aDNA sequences [see 20].

To estimate a substitution rate based on the cave-brown bear split, five cave bear sequences covering the same mitochondrial fragment were obtained from GenBank and aligned with five randomly-selected brown bear sequences. Bayesian phylogenetic analyses were performed using *BEAST*, using the HKY substitution model and a constant-size coalescent prior on the tree. To estimate an externally-calibrated substitution rate, the age of the cave-brown bear split was fixed to a value of 1.45 Myr in accordance with fossil evidence [64], [65]. Samples from the posterior were drawn every 20,000 MCMC steps over a total of 20,000,000 steps, with the first 10% discarded as burn-in.

Two molecular clock analyses were then performed on the alignment of 107 brown bears, using the model settings described above. In the first analysis, the externally-calibrated rate was used to place a normally-distributed prior (mean and standard deviation of 4.84×10^−3^ and 8.05×10^−4^ subs/site/Myr, respectively) on the substitution rate. In the second analysis, rate estimates were calibrated using the radiocarbon ages of the 51 ancient samples. Other details of the MCMC analysis were as described above. All input files are available upon request from the corresponding author.
